# *Cgl2* plays an essential role in cuticular wax biosynthesis in cabbage (*Brassica oleracea* L. var. *capitata*)

**DOI:** 10.1186/s12870-017-1162-8

**Published:** 2017-11-28

**Authors:** Dongming Liu, Jun Tang, Zezhou Liu, Xin Dong, Mu Zhuang, Yangyong Zhang, Honghao Lv, Peitian Sun, Yumei Liu, Zhansheng Li, Zhibiao Ye, Zhiyuan Fang, Limei Yang

**Affiliations:** 1 Key Laboratory of Biology and Genetic Improvement of Horticultural Crops, MOA, the Institute of Vegetables and Flowers, Chinese Academy of Agricultural Sciences, Beijing, 100081 China; 2 Key Laboratory of Horticultural Plant Biology, MOE, Key Laboratory of Horticultural Crop Biology and Genetic Improvement, MOA, Huazhong Agricultural University, Wuhan, 430070 China

**Keywords:** Cabbage, Glossy trait, Cuticular wax, Gene mapping, Functional analysis

## Abstract

**Background:**

The aerial parts of most land plants are covered with cuticular wax which is important for plants to avoid harmful factors. There is still no cloning study about wax synthesis gene of the alcohol-forming pathway in *Brassica* species.

**Results:**

Scanning electron microscopy (SEM) showed that, compared with wild type (WT), wax crystal are severely reduced in both the adaxial and abaxial sides of cabbage (*Brassica oleracea* L. var. *capitata* L.) leaves from the LD10GL mutant. Genetic analysis results revealed that the glossy trait of LD10GL is controlled by a single recessive gene, and fine mapping results revealed that the target gene *Cgl2* (*Cabbage glossy 2*) is located within a physical region of 170 kb on chromosome 1. Based on sequence analysis of the genes in the mapped region, the gene designated *Bol013612* was speculated to be the candidate gene. Gene *Bol013612* is homologous to *Arabidopsis CER4*, which encodes fatty acyl-coenzyme A reductase. Sequencing identified a single nucleotide substitution at an intron/exon boundary that results in an insertion of six nucleotides in the cDNA of *Bol013612* in LD10GL. The phenotypic defect of LD10GL was confirmed by a functional complementation test with *Arabidopsis* mutant *cer4*.

**Conclusions:**

Our results indicated that wax crystals of cabbage mutant LD10GL are severely reduced and mutation of gene *Bol013612* causes a glossy phenotype in the LD10GL mutant.

**Electronic supplementary material:**

The online version of this article doi: (10.1186/s12870-017-1162-8) contains supplementary material, which is available to authorized users.

## Background

Cabbage leaves usually appear glaucous due to the existence of cuticular wax crystals, but they tend to be glossy green in wax-deficient mutants. Wax plays important roles in protecting plants against various biotic and abiotic stresses, including drought, cold, UV radiation, insect attack, and pathogen infection [1–7]. Both the amount and composition of plant cuticular wax can differ across species, ontogeny, and environment [8–11]. The major compositions of cuticular wax are very long-chain fatty acids (VLCFAs) (C20-C40) and their derivatives, such as alkanes, aldehydes, ketones, esters, primary alcohols, and secondary alcohols [12].

In *Arabidopsis*, wax biosynthesis begins with the elongation cycle, which converts fatty acids (C16-C18) into VLCFAs by transferring a C2 unit from malonyl-CoA to long-chain acyl-CoA [13–17]. The generated VLCFAs are subsequently modified by the alcohol-forming pathway (also known as the acyl reduction pathway) or the alkane-forming pathway (also known as the decarbonylation pathway) to produce the main components of cuticular wax, including primary alcohols, esters, secondary alcohols, aldehydes, alkanes, and ketones [18, 19]. In *Arabidopsis*, primary alcohols and wax esters, the main products of the alcohol-forming pathway, account for 10–15% of the total wax on the stems and 15–25% on the leaves, while the alkane-forming pathway produces the majority of wax compounds on the stems (84%) and on the leaves (60%) [20].

With *cer* (*eceriferum*) and *gl* (*glossy*) mutants in *Arabidopsis* and other plants, several genes involved in wax biosynthesis have been isolated. These genes encode various proteins, including wax synthesis-related enzymes (*CER4*, *CER6*, *CER10*, FATB, and *GL8*) [20–24], wax transporters (*CER5* and *WBC11*) [25–27], and transcription factors (*SHN1*, *MYB30*, *MYB96*, and *WXP1*) [28–31]. In *Arabidopsis*, *CER4* encodes the fatty acyl-coenzyme A reductase (FAR), which is involved in production of VLCFAs and synthesis of primary alcohols [20].

Some genes involved in wax biosynthesis of *Brassica* species have been reported in recent studies. For example, gene *BrWax1* in *Brassica rapa* was mapped to an 86.4-kb physical interval on linkage group A1, and gene *Bra013809*, which is orthologous to the *CER2* gene in *Arabidopsis*, was predicted to be the candidate gene [32]. In *Brassica napus*, the *BnaA.GL* gene was fine-mapped using cDNA microarray chip analysis [33]. In cabbage, *Cgl1* was fine-mapped, and a candidate gene orthologous to *CER1* was identified [34]. Although several genes involved in wax biosynthesis have been identified in *Brassica* species, few genes of the alcohol-forming pathway have been cloned. In the present study, with help of wax-deficient cabbage mutant LD10GL, a high-resolution genetic map was constructed. The *Bol013612* gene was predicted to be the candidate gene associated with the glossy phenotype, and the prediction was proved by gene sequencing and functional analysis results. Based on the homology analysis with *Arabidopsis CER4* which encodes fatty acyl-coenzyme A reductase, *Bol013612* was speculated to be involved in the alcohol-forming pathway in cabbage. These results will help to illustrate the alcohol-forming pathway and accelerate research concerning wax metabolism in *Brassica* species.

## Results

### Wax is significantly reduced in LD10GL

**Fig. 1:**
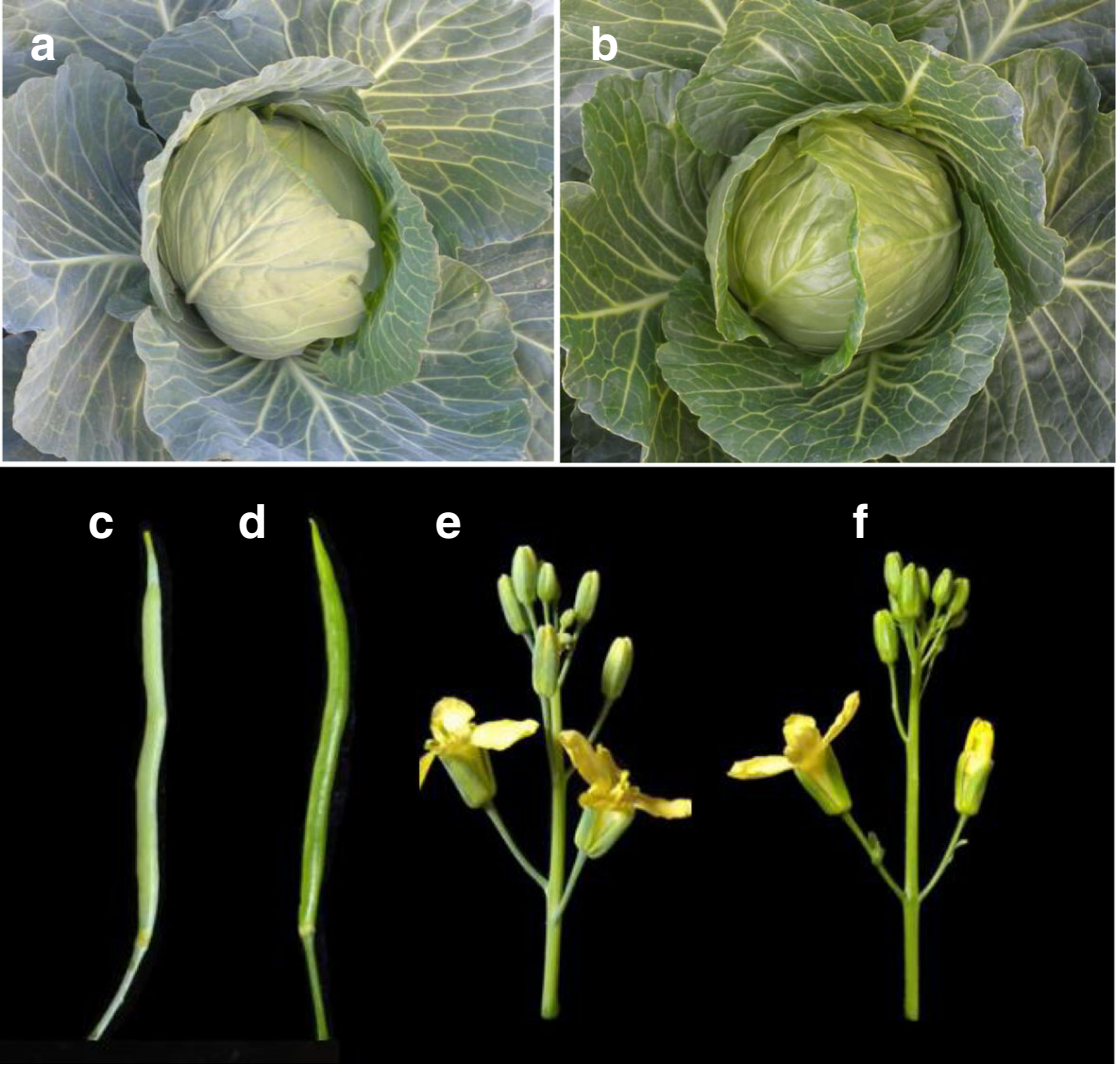
The morphological character of WT and LD10GL. **(a)** leaf, **(c)** seedpod, and **(e)** flower buds and stem of WT; **(b)** leaf, **(d)** seedpod, and **(f)** flower buds and stem of mutant LD10GL

WT (LD10) material is a high-generation cabbage (*B. oleracea* L. var. *capitata*) inbred line with glaucous appearance, and it is the near-isogenic line (NIL) of LD10GL. Surfaces of all the aerial organs of WT, such as the leaves (Fig. 1a), seedpods (Fig. 1c), stems, and flower buds (Fig. 1e), were glaucous with cuticular wax. In contrast, appearance of the corresponding organs tended to be glossy green in LD10GL (Fig. 1b, d and f).

**Fig. 2:**
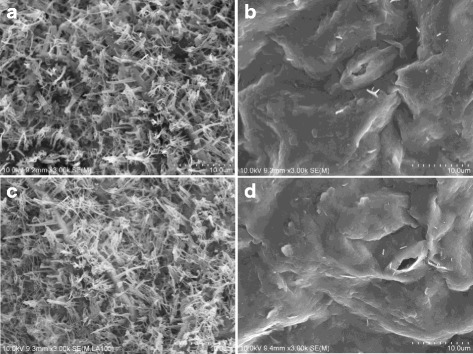
SEM images of wax crystals on the surface of cabbage leaves at 3000 × magnification. Appearance of leaf adaxial side of WT **(a)** and LD10GL **(b)**, and appearance of leaf abaxial side of WT **(c)** and LD10GL **(d)**

To study microscopic character of wax crystals, SEM was used to observe the adaxial and abaxial sides of the leaves. Result showed that both leaf sides of WT are covered with compact wax crystals and the crystals are rod shaped (Fig. 2a and c). In contrast, the amount of wax crystals in both the adaxial and abaxial sides of LD10GL was markedly reduced, and the wax crystals tended to be rodlet shaped (Fig. 2b and d).

**Fig. 3:**
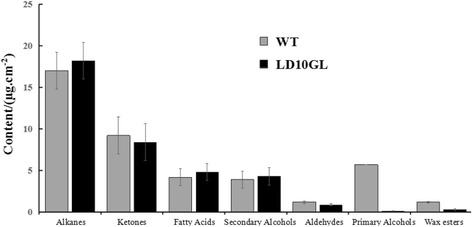
Cuticular wax compositions of WT and LD10GL. Three replicates were performed and the bars indicate SE of the mean

To dissect chemical components changes in LD10GL, wax composition of WT and LD10GL was analyzed by GC-MS. As shown in Fig. 3 and Additional file 1, similar content of alkanes, ketones, fatty acids, aldehydes, and secondary alcohols was observed by WT and LD10GL. But significant reduction of primary alcohols and wax esters was detected in LD10GL compared with WT. For example, the primary alcohol and wax ester content is about sixtyfold and fourfold in WT of LD10GL.

### The glossy green character of the LD10GL mutant is controlled by a single recessive gene

To investigate the inheritance of the glossy green trait in LD10GL, seeds of the parental materials, LD10GL(*B. oleracea* L. var. *capitata*)and M36 (*B. oleracea* var. alboglabra), and the derived F1, F2, BC1 and BC2 populations were sown. The glossy green individuals were identified visually. Results showed that surface of leaves and stems of all F_1_ plants is glaucous, suggesting that the glossy green trait is recessive to the glaucous trait. In the F_2_ population, 2130 of 2834 individuals showed a glaucous appearance, and 704 individuals showed a glossy green phenotype. The segregation ratio was confirmed to be 3:1 (χ2 = 0.24 < χ2_0.05_ = 3.84, *P* > 0.05) by the Chi-square test. In BC_1_ population, which was obtained through backcross of F_1_ with LD10GL plants, the numbers of glaucous plants and glossy plants were 202 and 192, respectively, displaying a ratio of 1:1 (χ2 = 0.03 < χ2_0.05_ = 3.84, *P* > 0.05). Furthermore, surface of all BC_2_ plants, which were achieved through backcrossing the F_1_ plant with M36, was glaucous (Table 1). All results above indicate that the glossy green trait of LD10GL is controlled by a single recessive gene.

**Table 1:** Segregation of glossy traits and the χ2 goodness-of-fit test of segregation in BC_1_ and F_2_ populations

Population	Total	Non-glossy	Glossy	Segregation ratio	χ2
P_1_(LD10GL)	16	0	16	–	–
P_2_(M36)	16	16	0	–	–
F_1_	100	100	0	–	–
F_2_	2834	2130	704	2.99:1	0.03
BC_1_(F_1_ × LD10GL)	394	202	192	1.05:1	0.24
BC_2_(F_1_ × M36)	268	268	0	–	–

### Preliminary genetic mapping of *Cgl2*

**Fig. 4:**
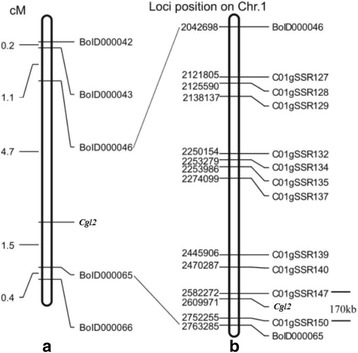
Genetic and physical maps of gene *Cgl2*. **a** Genetic linkage map constructed with the screened markers. **b** Physical map to fine map *Cgl2*

Two DNA pools were constructed by mixing equal amounts of DNA from 12 glossy green and 12 glaucous F_2_ individuals. To obtain the markers linked to gene *Cgl2*, 1064 pairs of primers were screened with the parents and DNA pools successively. As a result, only primer BoID000046 with polymorphism both between the parents and the two DNA pools was obtained. To further verify the linkage between BoID000046 and *Cgl2*, gDNA of 2834 F_2_ individuals was amplified as a template with primer BoID000046. As expected, genetic distance between primer BoID000046 and *Cgl2* was confirmed to be 4.7 cM (Fig. 4a). To acquire more markers linked to the *Cgl2* gene, another 200 pairs of primers on the same chromosome (chromosome C01) were analyzed with F_2_ individuals. Consequently, four more pairs of markers linked to *Cgl2* were obtained, and *Cgl2* was mapped to a genetic interval of 6.2 cM with markers BoID000046 and BoID000065 (Fig. 4a).

### Marker development and fine mapping of *Cgl2*

**Fig. 5:**
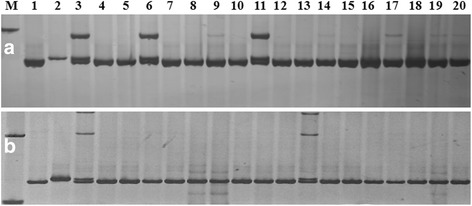
PCR amplification patterns of polymorphic markers. **a** C01gSSR147and (**b**) C01gSSR150 with the parents, F_1_, and the recessive individuals from the F_2_ population. The black arrows show the polymorphism between the glaucous and glossy green lines. *Lanes* 1, 2, and 3 is LD10GL, M36, and the F_1_ respectively. *Lanes* 4–20 represent the glossy green recessive individuals from the F_2_ populations

To narrow the mapped region, 50 pairs of new primers within the mapped region were developed according to *B. oleracea* genome database. Screened with the parental lines, 11 pairs of polymorphic primers were identified. These markers were used to screen 704 F_2_ individuals with the glossy phenotype for the recombinant plants. Based on the information of the polymorphic markers and the recombinants, a fine linkage map comprising 13 DNA markers was constructed (Fig. 4b). Gene *Cgl2* was delimited to a region between markers C01SSR147 and C01SSR150 with genetic distances of 0.2 cM and 0.1 cM, respectively, covering a physical distance of 170 kb (2503307–2,752,299) (Figs. 4b and 5a and b).

### Identification of the candidate gene for *Cgl2*

To identify the candidate gene, BLAST searches against the NCBI conserved domain database with genes in the mapped region were conducted. Result revealed that the gene *Bol013612* (C01:2,609,971:2,615,418), which contains the fatty acyl-coenzyme A reductase (FAR) domain, may be a candidate gene. The FAR enzyme usually acts on medium- and long-chain fatty acids and has been reported to be involved in the biosynthesis of plant primary alcohols and cuticular wax [20].

**Fig. 6:**
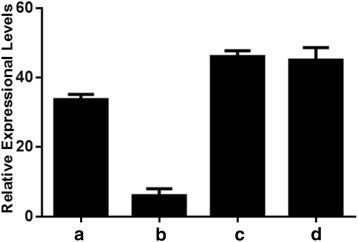
Expression level of gene *Bol013612* in the WT, LD10GL, cgl2-LD10GL, and Cgl2-LD10. The columns (**a**) and (**b**) are for expression of gene *Bol013612* in cabbage material WT and LD10GL; columns (**c**) and (**d**) depict expression levels of alleles *Bol013612* from LD10GL and WT when expressed in *Arabidopsis*. The date represents the means ± standard deviations of three replicates

**Fig. 7:**
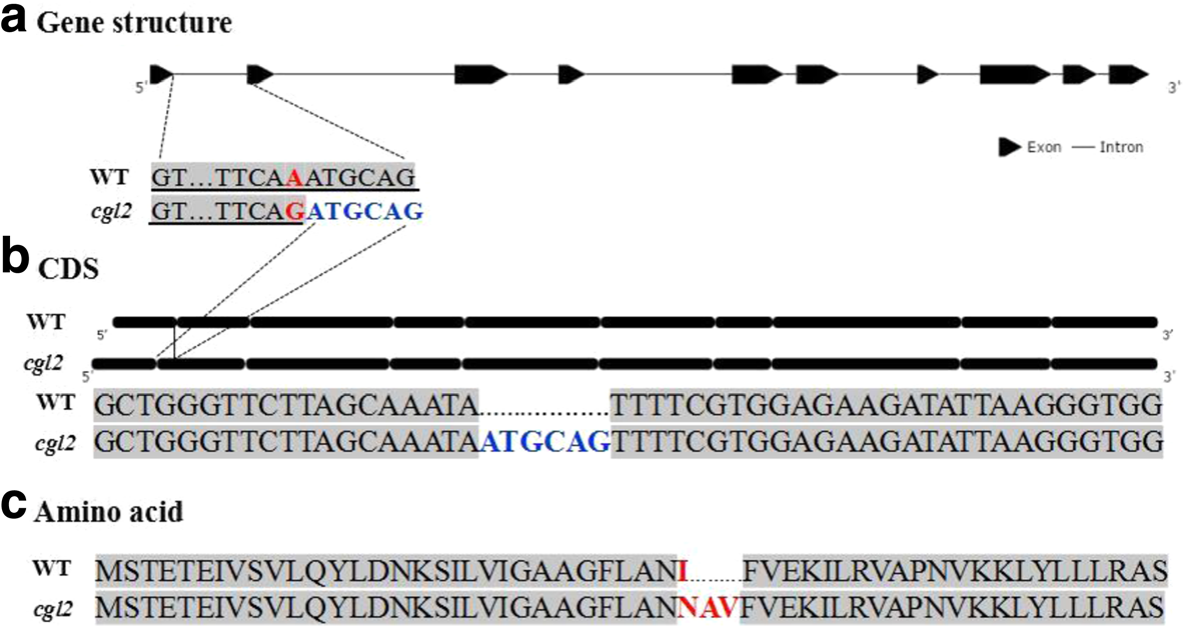
The gene structure and mutational pattern of gene *Bol013612* in LD10GL. **a** The gene structure and the nucleotide substitution in the gDNA of LD10GL. The underlined nucleotides are the partial sequence of the 1st intron. b The insertion of six nucleotides in gene *Bol013612* of LD10GL. **c** The mutational pattern in the level of amino acid of LD10GL

To examine transcriptional level of the candidate gene, expression of *Bol013612* was tested using qPCR. Results showed that the expression level in LD10GL is lower than that in WT, which may be induced by mutation and abnormal function of gene *Bol013612* in LD10GL (Fig. 6). The cDNA, gDNA, and nucleotide sequence approximately 2000 bp upstream of the gDNA of *Bol013612* were sequenced. Results showed that, compared with the WT, there exists a nucleotide substitution from adenine to guanine at nucleotide position 622 in the gDNA and an insert of six nucleotides in the cDNA of gene *Bol013612* (Fig. 7a and b; Additional file 2). According to the GT-AG rule, which implies that an intron should start with a ‘GT’ at the 5′ side and end with an ‘AG’ at the 3′ side [35], the mutational pattern in LD10GL was illustrated as follows: a single nucleotide substitution at position 622 in the gDNA brings a new terminal ‘AG’ to the first intron and thus generated an alternative 3′ splice site in intron 1. Because of the earlier ending of the first intron, the six additional nucleotides were included within the 5′ portion of exon 2 (Fig. 7a and b). As a result, six nucleotides are inserted into the cDNA sequence at nucleotide position 92 (Fig. 7b). Because of the insertion of the six nucleotides, one amino acid is modified and another two additional amino acids are introduced (Fig. 7c), which is just within the FAR domain of this protein (Additional file 3). The resulting change of amino acid sequence is considered to disrupt the gene function, influence the wax biosynthesis, and further lead to the glossy appearance.

### *Bol013612* can rescue the phenotype of the *Arabidopsis cer4* mutant

**Fig. 8:**
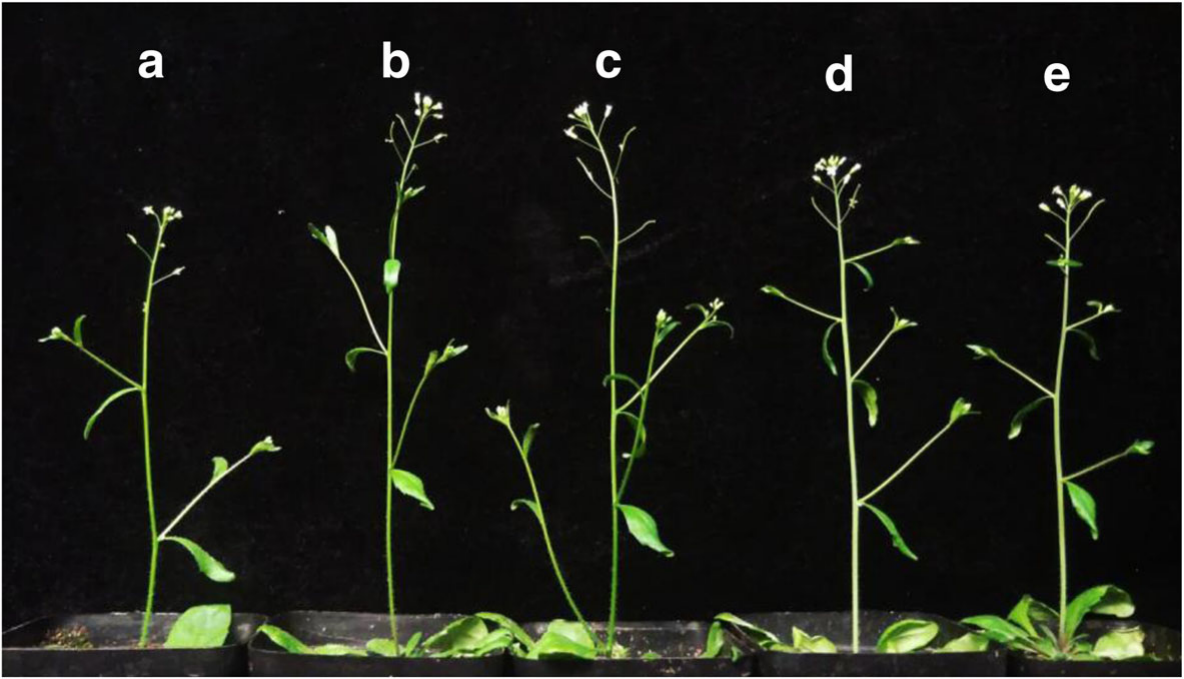
Recovery of cer4-Cgl2 plants from waxless *Arabidopsis* mutant *cer4*. Bright green appearance is presented by stems of *cer4* (**a**), cgl2-LD10GL (**b**), and *cer4*–3301(**c**); the color of stem in Cgl2-LD10 (**d**) turns to be glaucous, just like the WT (**e**)

**Fig. 9:**
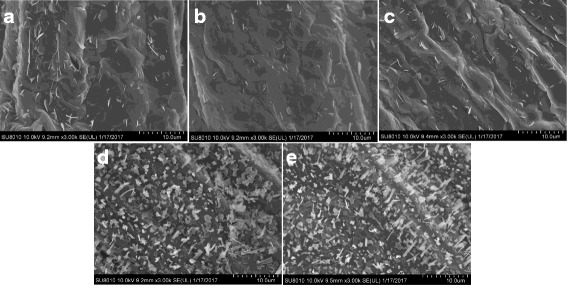
SEM images of wax crystals on the surface of *Arabidopsis* stems at 3000 × magnification. There are only a few wax crystals on the surface of *cer4* (**a**), cgl2-LD10GL (**b**), and *cer4*–3301 (**c**); There are amounts of wax crystals on the surface of Cgl2-LD10 (**d**), just like the WT (**e**), which indicates that the wax reduction defect was restored in Cgl2-LD10

Sequence analysis result show that protein sequence of *Bol013612* and *CER4* is highly homologous with each other (84.58%)(Additional file 4), and FAR domain was both contained by *CER4* and *Bol013612*. To further confirm the relationship between *Bol013612* and the glossy green phenotype in LD10GL, the full-length coding sequence (CDS) of the *Bol013612* gene from LD10GL was ectopically expressed in *Arabidopsis cer4* mutant under control of the 35S promoter (designated as cgl2-LD10GL). The *Arabidopsis cer4* plants, *cer4* plants with *Bol013612* cDNA from WT (designated as Cgl2-LD10), and mutant plants with an empty pCambia3301 vector (designated as cer4–3301) were used as the controls. Several independent transgenic lines were obtained from each process, and the *Bol013612* gene was ectopically expressed in *Arabidopsis* in the cgl2-LD10GL and Cgl2-LD10 plants (Fig. 6). As a result, the phenotypic defect of *Arabidopsis* mutant *cer4* was restored in Cgl2-LD10 (Fig. 8d), but the glossy appearance was still exhibited in cgl2-LD10GL (Fig. 8b) and cer4*–*3301 (Fig. 8c) plants; the wax load and morphology were restored to wildtype-levels in transgenic plants expressing Cgl2, but not cgl2-LD10GL (Fig. 9). The functional complementation test result and high sequence homology between gene *Bol013612* and known FAR-coding gene implicates protein Bol013612 is a wax biosynthesis enzyme. Collectively, these results illustrate that it is the mutation of *Bol013612* that influences wax biosynthesis and causes the glossy green trait in LD10GL.

## Discussion

Appearance of many plants is glaucous for the covering of cuticular wax, which constitutes the outermost barrier of plants. Compared with glaucous wild-type plants, most land plants with metabolic defects in wax biosynthesis are readily detectable with naked eye. With T-DNA insertion, many *Arabidopsis cer* (*eceriferum*) mutants were isolated and the corresponding genes were identified. Same as other *Arabidopsis cer* mutants, *cer4* has a glossy green appearance. But different from some sterile *cer* mutants, such as *cer1*, *cer3*, *cer6*, and *cer10*, *cer4* mutant is fertile [36].In contrast to the glaucous appearance in WT, surface of the leaves, seedpods, flower buds and stems is glossy green in cabbage LD10GL mutant. Changes of wax crystal density and shape were observed through SEM, indicating that it is the reduction of wax leads to glossiness in LD10GL. Similar as *cer4* mutant, cabbage mutant LD10GL is fertile, different from another cabbage glossy green mutant 10Q-961, in which few seeds were produced after self-pollination [34].

Plant wax compounds are produced through alcohol-forming pathway and the alkane-forming pathway in *Arabidopsis*. Many genes have been isolated. Several genes implicated in *Brassica* plants wax biosynthesis were reported in recent studies. Gene *BrWax1* in *Brassica rapa* was mapped to a fragment of 86.4 kb, and *Bra013809* was predicted to be the candidate gene for *BrWax1* [32]. The *BnaA.GL* gene in *Brassica napus* was proved to be located at the end of the A9 chromosome [33]. In cabbage, the *BoGL1* gene was delimited to the end of chromosome C08 by flanking marker SSRC08–76 with genetic distance of 0.2 cM and *Bol018504* was selected as the candidate gene [34]. Although several wax biosynthesis-related genes have been identified in *Brassica* species, little research about the alcohol-forming pathway and the related genes were reported.

In *Arabidopsis*, FAR catalyzes the two-step reduction of VLCFAs to produce primary alcohols. The primary alcohols together with C16:0 acyl-CoAs combine to produce alkyl esters. Primary alcohols and alkyl esters are two of the main cuticular wax components [20, 37]. *CER4* is one of the key genes encoding FAR in *Arabidopsis*. For reduction of primary alcohols and wax esters, glossy wax-deficient trait is displayed in *cer4* [20]. High level of protein sequence homology is exhibited between cabbage gene *Bol013612* and *CER4*. Similar as *cer4*, primary alcohol and wax esters are the main reduced wax compositions in mutant LG10GL.

As helpful material for metabolic mechanism research, the cabbage mutant LD10GL was applied to the wax biosynthesis study. Using a standard molecular genetic mapping strategy, the *Cgl2* gene was delimited to the interval between markers C01SSR147 and C01SSR150. Based on sequence analysis results, gene *Bol013612* was speculated to be the candidate gene. Concerning sequence homology and similar reduced wax composition in LD10GL and *cer4*, gene *Bol013612* was speculated to encode FAR. Linkage relationship of the glossy character in LD10GL and *Bol013612* was further confirmed by a functional complementation test. All results provided evidence that *Bol013612* may be the target gene for *Cgl2*.

All of these results will help us to further illustrate the formation of the glossy trait in LD10GL and wax biosynthesis in cabbage. More research needs to be carried out in future work, such as the metabolic mechanism of cabbage wax biosynthesis and identification of other genes involved.

## Conclusions

We fine-mapped a wax synthesis gene using the F_2_ populations, analyzed the genes present in the candidate region, and identified *Bol013612*, which is homologous to CER4, as a candidate gene for *Cgl2*. A 6-bp insertion resulted in the abnormal function of the FAR domain in the LD10GL glossy mutant line. The identification of *Cgl2* gene will enhance our understanding about its role in cabbage wax biosynthesis and lays a foundation for further study of cabbage wax metabolism.

## Methods

### Plant materials and genetic study

Plant materials in this study are all maintained in our lab. F_1_, F_2_, BC_1_, and BC_2_ populations were constructed using the female LD10GL parent (P_1_) and male M36 parent (P_2_) for inheritance and fine mapping studies. The glossy cabbage mutant LD10GL is a natural glossy mutant of LD10 cabbage. The gene responsible for the glossy green trait in LD10GL is named as *Cgl2* for the reason that *Cgl2* is the 2nd gene which was fine-mapped and cloned in cabbage (the 1st gene fine-mapped and cloned was named *Cgl1*). LD10 is a high-generation cabbage (*B. oleracea* L. var. *capitata*) inbred line with glaucous appearance and is referred as WT in this study. Same agronomic traits, such as shape of the head and plant type are exhibited by LD10 and LD10GL, except the glossy green appearance. M36 is a Chinese kale (*Brassica oleracea var. alboglabra*) material with glaucous appearance. F_1_ plants were obtained by crossing LD10GL and M36. One F_1_ plant was used as the female parent material in the backcrosses with LD10GL and M36, and F_2_ plants were obtained by selfing the same F_1_ plant. All the plant materials were provided by the Cabbage and Broccoli Research Group, Institute of Vegetables and Flowers (IVF), Chinese Academy of Agricultural Sciences (CAAS) and grown in the greenhouse in Changping (39°54′N, 116°13′E, Beijing, China) according to local crop management practices. The glossy phenotype was assessed and recorded at the five-leaf stage. The segregation ratios of the F_2_ and BC_1_ populations were analyzed with the Chi-square test (χ^2^).

### SEM and GC-MS analysis

Fresh leaves from the five-leaf-stage plants were fixed overnight in 2% glutaraldehyde, mounted on specimen stubs using double-sided tape and then coated with gold particles in a SEMPrep2 sputter coater (Nanotech). The phenotype was analyzed by SEM (S-4800, Hitachi, Japan) with a secondary electron detector at a high voltage (10 kV). **GC-MS** analysis of cuticular wax was carried out as described as Tang [38].

### The mapping strategy

Total genomic DNA from fresh leaves was extracted with the cetyltrimethylammonium bromide (CTAB) protocol, and the concentration of genomic DNA was adjusted to 30 ng/μl [39]. Polymerase chain reaction (PCR) and polyacrylamide gel electrophoresis (PAGE) was performed with 20 μl of reaction mixture [40]. Using the bulked segregant analysis (BSA) method, 12 glaucous and 12 glossy individuals from the F_2_ population were selected to construct two DNA bulks. One thousand two hundred sixty-four pairs of SSR primers that were designed according to data of the *B. oleracea* genome database (*Brassica* Database, BRAD; http://brassicadb.org/brad/index.php) [41] were screened by the parental materials and two DNA pools successively for mapping analysis. The linkage relationships between the *Cgl2* gene and the polymorphic primers were confirmed with F_2_ individuals. Linkages between markers and the *Cgl2* gene were determined using the software Joinmap 4.0 (Kyasma, NL). To finely map *Cgl2*, 50 pairs of SSR primers near the preliminary mapping region were developed and used to screen for the recombinants among 704 recessive F_2_ individuals. The individuals whose gel bands were the same as those of M36 or the F_1_ were used as the recombinants and selected for further analysis. The linkage between the markers and the *Cgl2* gene was determined with the Kosambi mapping function, and a genetic map was constructed using the software Mapdraw 2.1 [42, 43]. The sequences of all polymorphic markers used in this study are listed in Table 2.

**Table 2:** Sequences of primers

Primer name	Forward primer sequence (5′-3′)	Reverse primer sequence (5′-3′)
BoID000042	ACCATGTTCATAGCAAGCTC	ACACTCGCACGTAAAGAAAT
BoID000043	GCTGATGGAAGGAAGATGTA	CCTGCTTTGAAAATTCACTC
BoID000046	CACAAAATCATTAGGCCAAC	AAAAGACAGTGCCTTCCTAA
BoID000065	ATTGGTTTAGTGCAAGTGCT	CTTCATTTTTCGAGGTCAAG
BoID000066	TCCTGTGATGTGATTTTCCT	AAAATGCTTGTGAAGTGACC
C01gSSR127	GGCGTGAGACAGTCCAAT	TGAGAACCTTGCTTACAAAC
C01gSSR128	TCGTTCGACTGTATTTGACA	CAGTGTGTTGATGATGAAGC
C01gSSR129	ATGCATCATGTTCGTTACTG	TCACTGGTAAATGATGGGTT
C01gSSR132	CAAAACATGAGACCAAGACC	GCTGGATAGCATTCTAAAGG
C01gSSR134	CAAACAATGTGAGGGAAGTT	GTATGTTCTTGTTGGGTTGG
C01gSSR135	CACAACTTATTCGCTGACAA	CACTCTTATTACGTGCTCCA
C01gSSR137	GTTCAGTCTGGATTTTGCTT	TCCAAAGACGAACAAAAGAG
C01gSSR139	TTAAGGGATTTTGGACAGG	GAAGGATATACTGTGGTGGC
C01gSSR140	CCCACTTCACTCTGCTTATG	GTATGGTCGAAGTGGTATGC
C01gSSR147	TAAAAATGAGGAAGTGGCCG	GGGCAGATAGTCTAGCACGC
C01gSSR150	AGAATTCAAGTCTTTGCGAG	TATCCCCTGTATGGTGATGT
Bol013612g1	ACTAGGCATAATGTGTGCG	CACGACAGTCATCAGAAGC
Bol013612g2	GTTTGGACTTGTAGTGCTTCTG	GATTATCTATGGGTGGTAACGG
Bol013612g3	AGACGAGGTTTGTCCCTTC	CATACTCTTCTAAAGCACCCG
Bol013612g4	TATCAACACACTGGGCGTC	ACACGAACCACCTTACCTTC
Bol013612g5	GGCTCACCAGGTTCTATTCC	CCCAACTTCCCAATCAGAC
Bol013612c1	CTCGCTCATACGTACATAC	TTTAAATAGCAACATCCC
qPCR-013612	AGCCTCTCCTGAAACCATC	CGGACGAATCAACACAAG
qPCR-actin-Bol	CCTCCGTCTTGACCTTGC	GTCTCCATCTCCTGCTCGT

### Identification and sequence analysis of the candidate gene

Conserved domains of genes between the two closest markers were analyzed using the BLAST program (http://www.ncbi.nlm.nih.gov/Structure/cdd/wrpsb.cgi) [44]. Genes with wax-related conserved domains were selected as the candidate genes. The level of protein sequence homology between *Bol013612* and *Arabidopsis CER4* was analyzed with software MultAlin (http://multalin.toulouse.inra.fr/multalin/multalin.html) [45] and DNAMAN software (http://www.lynnon.com/dnaman.html). The expression level of the candidate gene in WT and LD10GL was tested by qPCR. Sequences of the candidate genes were amplified using the gDNA and cDNA as templates with a KAPPA HiFi HotStart ReadyMix PCR Kit (Kappa Biosystems). The PCR products were sequenced by BGI TechSolutions Co., Ltd. Sequences were aligned with the software MultAlin (http://multalin.toulouse.inra.fr/multalin/multalin.html) [45]. The gene structure was analyzed with the Gene Structure Display Server software (http://gsds.cbi.pku.edu.cn/index.php) [46].

The primers used to amplify the gDNA sequence were designed according to the *B. oleracea* genome database (*Brassica* Database, BRAD; http://brassicadb.org/brad/index.php) [41] with the software Primer Premier 5 [47] and were named *Bol013612*g1, *Bol013612*g2, *Bol013612*g3, *Bol013612*g4, and *Bol013612*g5 (Table 2). The primers used to amplify the cDNA sequence were designed according to the *B. oleracea* transcriptome database (http://www.ncbi.nlm.nih.gov/geo/query/acc.cgi?acc=GSE42891) [41] and named *Bol013612*c1 (Table 2). Total RNA extraction, first-strand cDNAs synthesis, and real-time PCR were performed as previously reported [34]. Sequences of the primers for qPCR named qPCR-013612 and qPCR-actin-Bol are listed in Table 2. Each sample was tested in triplicate.

### Transformation of *Arabidopsis* mutants

The cDNA of *Bol013612* from LD10GL and WT was cloned and inserted into the pCAMBIA3301 vector between the *Nco*I and *Bst*EII sites. The constructed vectors contained the 35S CaMV promoter and a Basta resistance marker. The plasmid was introduced into *Agrobacterium* EHA105 via heat shock and transformed into *Arabidopsis* plants using the floral dip method [48]. *Arabidopsis thaliana* mutant material *cer4* (ecotype Columbia; named SALK_000575C in Arabidopsis Biological Resource Center, ABRC), which has glossy green stem surface, was used for experiments involving transformation [49]. Seeds of transformed *Arabidopsis* plants were selected on MS (Murashige and Skoog) medium containing 50 mg/ml of Basta. qRT-PCR was employed to further confirm the successful transfer of 35S:Bol013612 in the stems with the primers qPCR-013612 and qPCR-actin-At (Table 2).

## Additional files

Additional file 1: Cuticular wax composition of WT and LD10GL. (DOCX 20 kb)

Additional file 2: CDS of gene *Bol013612* in WT and LD10GL. (DOCX 21 kb)

Additional file 3: Insertion of six nucleotides at nucleotide position 92 in cDNA sequence of gene *Bol013612* is just within the FAR domain of this protein. (DOCX 106 kb)

Additional file 4: Protein sequence homology analysis of gene *Bol013612* and *CER4. (DOCX 32 kb)*

## Abbreviations

ABRC: Arabidopsis Biological Resource Center
*CER*: *Eceriferum*
Cgl2: Cabbage glossy 2
FAR: Fatty acyl-coenzyme A reductase
gl: glossy
MOA: Ministry of agricultural
MOE: Ministry of education
MS: Murashige and Skoog
SEM: Scanning electron microscopy
UV: Ultravioletray
VLCFAs: Very long-chain fatty acids
WT: Wild type
