# Supplementary material for: Cgl2 plays an essential role in cuticular wax biosynthesis in cabbage (Brassica oleracea L. var. capitata)
Source: BMC Plant Biol. 2017 Nov 28;17:223. doi: 10.1186/s12870-017-1162-8 (PMC5704555; doi:10.1186/s12870-017-1162-8)
Supplement: Supplementary file 1 — Cuticular wax composition of WT and LD10GL. (DOCX 20 kb) [file 12870_2017_1162_MOESM1_ESM.docx]

**Additional file 1 Cuticular wax compositions of WT and LD10GL (µg/cm^2^).**

| Line | Alkanes | Ketones | Fatty Acids | Secondary Alcohols | Aldehydes | Primary Alcohols | Wax Esters |
| --- | --- | --- | --- | --- | --- | --- | --- |
| WT | 17.1±2.3 | 9.2±1.2 | 4.2±0.7 | 3.9±0.9 | 1.2±0.4 | 5.7±1.3 | 1.2±0.5 |
| LD10GL | 18.2±2.2 | 8.4±2.2 | 4.8±1.0 | 4.3±1.1 | 0.9±0.2 | 0.1±0.0 | 0.3±0.1 |
