# Supplementary material for: Cgl2 plays an essential role in cuticular wax biosynthesis in cabbage (Brassica oleracea L. var. capitata)
Source: BMC Plant Biol. 2017 Nov 28;17:223. doi: 10.1186/s12870-017-1162-8 (PMC5704555; doi:10.1186/s12870-017-1162-8)
Supplement: Supplementary file 2 — CDS of gene Bol013612 in WT and LD10GL. (DOCX 21 kb) [file 12870_2017_1162_MOESM2_ESM.docx]

**Additional file 2 CDS of gene *Bol013612* in WT**

ATGTCGACAGAAACGGAGATCGTGAGTGTTCTTCAGTACCTTGACAACAAATCCATATTGGTCATTGGAGCTGCTGGGTTCTTAGCAAATATTTTCGTGGAGAAGATATTAAGGGTGGCACCTAACGTGAAGAAACTCTATCTTCTTCTAAGAGCATCAACTGAAAAATCTGCTACCCAGAGGTTTAAAGACGAGATTTTAGGGAAGGACTTGTACAGGGTACTGAAGGAGAAGTATGGTCCAAATCTAAATCAACTTACATCTGAGAAAGTTACTGTTGTCAGTGGGGACATTTCCCTTGAGGATCTGGGTCTTCAAGACACTGACTTGGAACATGAGATGATCCACCAAGTTGATGCCATTGTTAATTTAGCTGCAACTACTAAATTTGATGAAAGATACGATATAGCACTTGGTATCAACACACTGGGCGTCCTCAATGTCTTGAATTTCGCCAAGAGATGTGCAAAGATTAATATTTTTGTTCAAGTATCAACAGCTTACGTTTGCGGGGAAAAATCTGGTTTGATAATGGAAACACCATACCGTATGGGTGAGACGTTGAATGGAACCACCGGCCTAGACATCAACCATGAGAAGAAATTGGTCGAGGAGAAACTTGACCAGCTCCGTGTCACCGAAGCCTCTCCTGAAACCATCACTCAAACCATGAAAGATATGGGGCTCACCAGGGCAAGAACGTATGGATGGCCAAACACTTACGTTTTCACAAAAGCAATGGGAGAGATGATTGTAGGGGCAAAAAGGGGGAATTTACCACTTGTGTTGATTCGTCCGTCAATTATTACTAGCACTATCAAAGAACCATTCCCTGGCTGGACCGAAGGCATCAGGACCATTGATACTCTAGGTGTCGGATATGGTAAGGGCAGACTCACATGCTTCCTTGGTGATCTTAATGCTGTTTCCGATGTGATGCCAGCAGATATGGTAGTAAATTCGATGTTAGTGTCGATGGCTGTTCAAGCTGGAAAACAGAAAGAAACTATTTATCATGTGGGTTCCTCGCTAAGAAATCCCTTGAAGAATGAGAAACTTCCTGAGATAGCATACCATTGTTTTACTACCAAACCATGGACTAACAAAGAAGGGAAGGTGGTTCGTGTAAAGAATATCGAGATTCTGAGTTCTATGGCTAGTTTCCACAGATACATGGCCATACATTACTTGATCCCATTAAAGGGACTTGCATTATTAAACATTGTATTATGCAAGCTTTTGGACAAAAGTTTGAAGGATTTTCATAGGAAGATAAACTTTGCATTCCGGCTCGTTGAACTTTACCAGCCCTACCTCTTTTTCAATGGAGTATTTGATGATACAAACACGGAAAAGCTGCAAGGAATTGTGTTGAAGACAGAAGCCGAAACCGAGATGTTCTGTTTTGATCCAACAGTTATCAATTGGGACGACTATTTTGTGGATATACATGTTCCTGGACTGGTTAAGTACGTTTTCTAA

**CDS of gene *Bol013612* in LD10GL**

ATGTCGACAGAAACGGAGATCGTGAGTGTTCTTCAGTACCTTGACAACAAATCCATATTGGTCATTGGAGCTGCTGGGTTCTTAGCAAATAATGCAGTTTTCGTGGAGAAGATATTAAGGGTGGCACCTAACGTGAAGAAACTCTATCTTCTTCTAAGAGCATCAACTGAAAAATCTGCTACCCAGAGGTTTAAAGACGAGATTTTAGGGAAGGACTTGTACAGGGTACTGAAGGAGAAGTATGGTCCAAATCTAAATCAACTTACATCTGAGAAAGTTACTGTTGTCAGTGGGGACATTTCCCTTGAGGATCTGGGTCTTCAAGACACTGACTTGGAACATGAGATGATCCACCAAGTTGATGCCATTGTTAATTTAGCTGCAACTACTAAATTTGATGAAAGATACGATATAGCACTTGGTATCAACACACTGGGCGTCCTCAATGTCTTGAATTTCGCCAAGAGATGTGCAAAGATTAATATTTTTGTTCAAGTATCAACAGCTTACGTTTGCGGGGAAAAATCTGGTTTGATAATGGAAACACCATACCGTATGGGTGAGACGTTGAATGGAACCACCGGCCTAGACATCAACCATGAGAAGAAATTGGTCGAGGAGAAACTTGACCAGCTCCGTGTCACCGAAGCCTCTCCTGAAACCATCACTCAAACCATGAAAGATATGGGGCTCACCAGGGCAAGAACGTATGGATGGCCAAACACTTACGTTTTCACAAAAGCAATGGGAGAGATGATTGTAGGGGCAAAAAGGGGGAATTTACCACTTGTGTTGATTCGTCCGTCAATTATTACTAGCACTATCAAAGAACCATTCCCTGGCTGGACCGAAGGCATCAGGACCATTGATACTCTAGGTGTCGGATATGGTAAGGGCAGACTCACATGCTTCCTTGGTGATCTTAATGCTGTTTCCGATGTGATGCCAGCAGATATGGTAGTAAATTCGATGTTAGTGTCGATGGCTGTTCAAGCTGGAAAACAGAAAGAAACTATTTATCATGTGGGTTCCTCGCTAAGAAATCCCTTGAAGAATGAGAAACTTCCTGAGATAGCATACCATTGTTTTACTACCAAACCATGGACTAACAAAGAAGGGAAGGTGGTTCGTGTAAAGAATATCGAGATTCTGAGTTCTATGGCTAGTTTCCACAGATACATGGCCATACATTACTTGATCCCATTAAAGGGACTTGCATTATTAAACATTGTATTATGCAAGCTTTTGGACAAAAGTTTGAAGGATTTTCATAGGAAGATAAACTTTGCATTCCGGCTCGTTGAACTTTACCAGCCCTACCTCTTTTTCAATGGAGTATTTGATGATACAAACACGGAAAAGCTGCAAGGAATTGTGTTGAAGACAGAAGCCGAAACCGAGATGTTCTGTTTTGATCCAACAGTTATCAATTGGGACGACTATTTTGTGGATATACATGTTCCTGGACTGGTTAAGTACGTTTTCTAA
