# Supplementary material for: Cgl2 plays an essential role in cuticular wax biosynthesis in cabbage (Brassica oleracea L. var. capitata)
Source: BMC Plant Biol. 2017 Nov 28;17:223. doi: 10.1186/s12870-017-1162-8 (PMC5704555; doi:10.1186/s12870-017-1162-8)

**Additional file 3** Insertion of six nucleotides at nucleotide position 92 in cDNA sequence of gene *Bol013612* is just within the FAR domain of this protein.


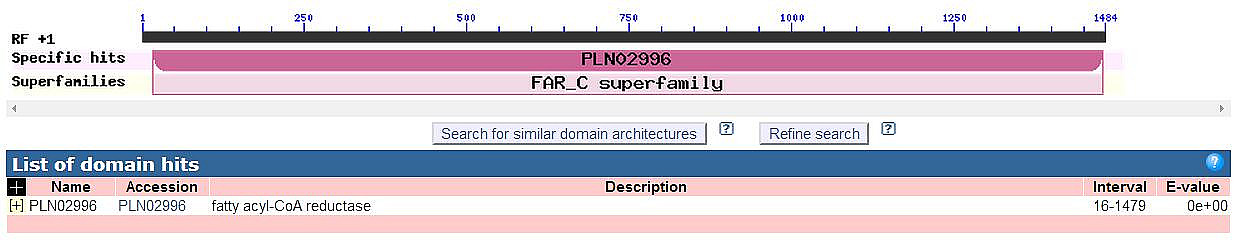

Supplement: Supplementary file 3 — Insertion of six nucleotides at nucleotide position 92 in cDNA sequence of gene Bol013612 is just within the FAR domain of this protein. (DOCX 106 kb) [file 12870_2017_1162_MOESM3_ESM.docx]
