# Supplementary figures and images for: Cgl2 plays an essential role in cuticular wax biosynthesis in cabbage (Brassica oleracea L. var. capitata)
Source: BMC Plant Biol. 2017 Nov 28;17:223. doi: 10.1186/s12870-017-1162-8 (PMC5704555; doi:10.1186/s12870-017-1162-8)

**Additional file 4 Protein sequence homology analysis of gene *Bol013612* and *CER4***


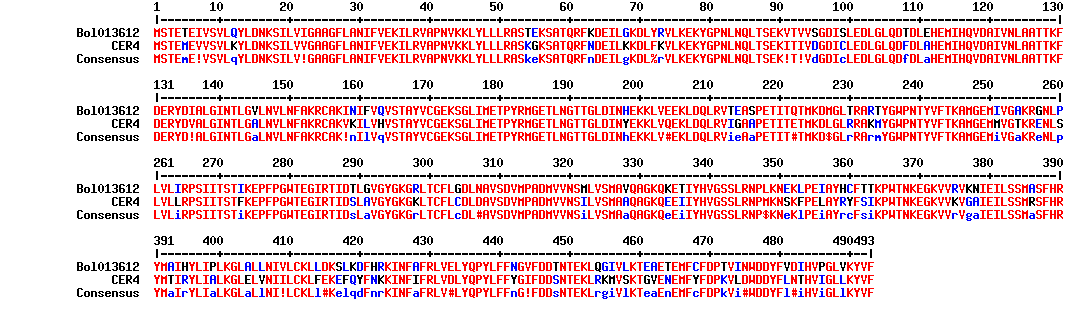

Supplement: Supplementary file 4 — Protein sequence homology analysis of gene Bol013612 and CER4. (DOCX 32 kb) [file 12870_2017_1162_MOESM4_ESM.docx]
